# Peripheral and central levels of kynurenic acid in bipolar disorder subjects and healthy controls

**DOI:** 10.1038/s41398-019-0378-9

**Published:** 2019-01-29

**Authors:** Carl M. Sellgren, Jessica Gracias, Oscar Jungholm, Roy H. Perlis, Göran Engberg, Lilly Schwieler, Mikael Landen, Sophie Erhardt

**Affiliations:** 1 Department of Physiology and Pharmacology, Karolinska Institutet, Stockholm, Sweden; 2 Center for Experimental Drugs and Diagnostics, Center for Genomic Medicine and Department of Psychiatry, Massachusetts General Hospital, Boston, MA USA; 3 Department of Psychiatry, Harvard Medical School, Boston, MA USA; 4 Stockholm County Council, Stockholm, Sweden; 5 Institute of Neuroscience and Physiology, Department of Psychiatry and Neurochemistry, The Sahlgrenska Academy, University of Gothenburg, Gothenburg, Mölndal Sweden

## Abstract

Metabolites of the kynurenine pathway of tryptophan degradation, in particular, the *N*-Methyl-d-aspartic acid receptor antagonist kynurenic acid (KYNA), are increasingly recognized as primary pathophysiological promoters in several psychiatric diseases. Studies analyzing central KYNA levels from subjects with psychotic disorders have reported increased levels. However, sample sizes are limited and in contrast many larger studies examining this compound in blood from psychotic patients commonly report a decrease. A major question is to what extent peripheral KYNA levels reflect brain KYNA levels under physiological as well as pathophysiological conditions. Here we measured KYNA in plasma from a total of 277 subjects with detailed phenotypic data, including 163 BD subjects and 114 matched healthy controls (HCs), using an HPLC system. Among them, 94 BD subjects and 113 HCs also had CSF KYNA concentrations analyzed. We observe a selective increase of CSF KYNA in BD subjects with previous psychotic episodes although this group did not display altered plasma KYNA levels. In contrast, BD subjects with ongoing depressive symptoms displayed a tendency to decreased plasma KYNA concentrations but unchanged CSF KYNA levels. Sex and age displayed specific effects on KYNA concentrations depending on if measured centrally or in the periphery. These findings implicate brain-specific regulation of KYNA under physiological as well as under pathophysiological conditions and strengthen our previous observation of CSF KYNA as a biomarker in BD. In summary, biomarker and drug discovery studies should include central KYNA measurements for a more reliable estimation of brain KYNA levels.

## Introduction

Increased concentration of kynurenic acid (KYNA), a neuroactive end-product of the kynurenine pathway of tryptophan degradation^1^, has repeatedly been observed in cerebrospinal fluid (CSF) and postmortem brain tissue of subjects with schizophrenia or bipolar disorder^2–9^. The kynurenine pathway is critically controlled by the immune system, and in vitro experiments have revealed that interleukin (IL)-1β as well as IL-6 induces the biosynthesis of KYNA in human astrocytes^6,10^, i.e., the main producer of KYNA in the brain^1^. Notably, increased CSF levels of IL-1β and IL-6 have also been observed in schizophrenia^10^, as well as IL-1β has in the CSF from bipolar disorder (BD) subjects^11,12^. Although based on limited sample sizes, CSF levels of KYNA in BD subjects, as well as IL-1β levels, have been reported to be selectively increased in subjects with a history of psychotic episodes^5,6,9^, and linked to persistent set-shifting impairment^6^. In line with these clinical associations, rodent studies have confirmed that KYNA causes disruption of pre-pulse inhibition^13^, as well as behavioral responses analogous to impaired set-shifting ability^14^. Although KYNA has established antagonistic actions on both the glycine co-agonist site of the *N*-methyl-d-aspartic acid receptor (NMDAR) and the cholinergic α7 nicotinic receptor^1^, the exact molecular mechanisms in vivo remain elusive.

While KYNA crosses the blood-brain barrier (BBB) poorly^15^, rodent studies suggest that approximately 60% of brain kynurenine, the precursor to KYNA, comes from peripheral sources^1^. However, to what extent peripheral sources of kynurenine influences brain KYNA levels, under physiological as well as pathophysiological conditions in humans, remains controversial^16^, as well-powered studies including intra-individual analyses of peripheral and central kynurenine metabolites are still lacking. BBB permeability for a certain compound may differ between species^17^, and depends on potential pathophysiological leaking of BBB. Assuming that the brain KYNA pool is connected to peripheral kynurenine levels, KYNA in brain may still be targeted by specific regulatory mechanisms that limit the use of peripheral KYNA measurements as a predictor of central KYNA levels. Studies of peripheral KYNA concentrations in BD and schizophrenia have reported conflicting results^18–20^. In BD, this could be a result of mood state at the time of sampling since increased central KYNA levels have so far exclusively been observed in euthymic patients^4–6^ and decreased peripheral levels in inpatients with an ongoing mood episode^19^, or euthymic patients pooled with mildly depressed patients^21^. Importantly, if blood levels of KYNA do reflect central levels, cumbersome CSF sampling could be avoided in clinical as well as in drug discovery studies.

Using a large cohort of systematically phenotyped BD type I/II subjects and matched healthy controls (HCs), randomly sampled from the normal population, we here examine intra-relationships and inter-relationships between peripheral and central KYNA levels as well as subgroup analyses of defined BD groups.

## Materials and methods

The study was approved by the institutional review board of the Karolinska Institutet. Informed consent was obtained from all included subjects.

### Study population

All patient data were collected from Swedish BD participants in a long-term follow-up program at a tertiary outpatient unit in Stockholm. A subset of the bipolar subjects (*n* = 76) and HCs (*n* = 46) have previously been analyzed with detection of increased CSF KYNA levels in psychotic bipolar subjects^6^, and other labs have performed plasma studies in bipolar disorder subjects with reports of altered KYNA levels using smaller sample sizes^18–20^. Thus, the current sample sizes were deemed to ensure adequate power. The diagnostic procedure has been outlined in detail previously^22,23^. Briefly, assessments are based on all available sources of information, including patient records, and interviews with next of kin when feasible. A consensus panel of experienced board-certified psychiatrists specialized in bipolar disorder made a “best estimate” diagnosis. Enrolled study subjects are at least 18 years of age, meet the Diagnostic and Statistical Manual of Mental Disorders 4th Edition (DSM-IV) criteria for a bipolar disorder I or II. Further, the Montgomery-Åsberg Depression Rating Scale (MADRS)^24^ and the Young Mania Rating Scale (YMRS)^25^ were used to assess the extent of ongoing depressive and manic symptoms in patients. The baseline clinical diagnostic instrument for BD used the Affective Disorder Evaluation (ADE)^26^, translated and modified to suit Swedish conditions after permission from the originator Gary S. Sachs. Co-morbid psychiatric disorders were collected using the Mini International Neuropsychiatric Interview (M.I.N.I.)^27^. Experienced psychologists performed the neuropsychological assessments, using the Delis–Kaplan Executive Function System (D-KEFS). To obtain a sensitive measure of set-shifting, we employed the TMT and extracted the total time taken for Combined Letter/Number Switching minus the Combined Number Sequencing + Letter Sequencing, that is, the “switching cost”. Raw contrast scores were transformed into age-corrected scaled contrast scores based on normative data in which an achievement score of 10 represents the mean in each age group^28^.

The general population HCs were randomly selected from the same catchment area by Statistics Sweden and underwent a similar clinical evaluation as the bipolar subjects.

Primary analyses were performed including all BD type I/II patients with available data. Sensitivity analyses were performed excluding patients with (1) autoimmune disorders, (2) heart disease (recent myocardial infarct or ongoing angina), (3) cerebrovascular disease, (4) diabetes, (5) multiple sclerosis, (5) Alzheimer’s disease, (6) Parkinson’s disease, (7) epilepsy, 8) brain tumor, or (9) migraine.

### Collection of cerebrospinal fluid and blood

Subjects fasted overnight before the standardized CSF and blood collection that occurred between 9.00 and 10.00 a.m. For CSF collection, a non-cutting spinal needle was inserted into the L3/L4 or L4/L5 interspace and a total volume of 12 mL of the CSF was collected, gently inverted to avoid gradient effects, and divided into 1.0–1.6 mL aliquots that were stored at −80 °C pending analysis.

### Analysis of kynurenic acid

CSF and plasma were analyzed for KYNA content using a High Performance Liquid Chromatography (HPLC) system with fluorescence detection as previously described^6^. To precipitate residual protein, samples were centrifuged at 20,000×*g* (5 min for CSF and 3 min for plasma). Supernatants from plasma samples were then also diluted with an equal volume of perchloric acid (0.4 M) and the centrifugation procedure was repeated and followed by addition of 70% perchloric acid at a volume equal to 1/7 of the new supernatant. Prior to analysis the plasma samples were then centrifuged a third time. This resulted in a dilution factor of 2.29, which was later on multiplied on to the resulting concentrations to receive the corresponding plasma concentration. After thawing and centrifugation the samples were then manually injected into the HPLC system (50 μL CSF or 20 μL plasma). The HPLC system included a dual-piston high-liquid delivery pump (Bischoff, Leonberg, Germany), a ReproSil-Pur C18 column (4 × 100 mm, Dr. Maisch GmbH, Ammerbuch, Germany) and a fluorescence detector (Jasco Ltd, Hachioji city, Japan) with an excitation wavelength of 344 nm and an emission wavelength of 398 nm (18 nm bandwidth). A mobile phase of 50 mM sodium acetate (pH 6.2, adjusted with acetic acid) and 7.0% acetonitrile was pumped through the HPLC-column at a flow rate of 0.5 mL/min. To enable fluorescent detection, zinc acetate (0.5 M) was delivered after the column by a piston pump P-500 (Pharmacia) at a flow rate of 10 mL/h. Signals from the fluorescence detector were transferred to a computer for analysis with Datalys Azur (http://datalys.net). The retention time of KYNA was about 7–8 min. The sensitivity of the system was verified by analysis of standard mixtures of KYNA with concentrations from 3.75 to 30 nM, which resulted in a linear standard plot. To verify the reliability of this method, samples were analyzed in duplicate, and the mean intra-individual variation was below 5%.

### Statistics

Correlation or partial correlation analyses were performed using Spearman’s correlation coefficients. Group analyses were performed using Mann–Whitney *U*-tests, logistic regression analyses, or Chi-square tests as indicated after confirming the assumptions of each test. All reported p-values are two sided. All analyses used the statistical software program R (version 2.7.0; https://www.r-project.org) and graphs were produced using Graph-Pad prism 6.0 (http://www.graphpad.com/) or the R package “plotly” (https://plotly-book.cpsievert.me).

## Results

### Peripheral and central KYNA levels in bipolar disorder and healthy controls

**Fig. 1:**
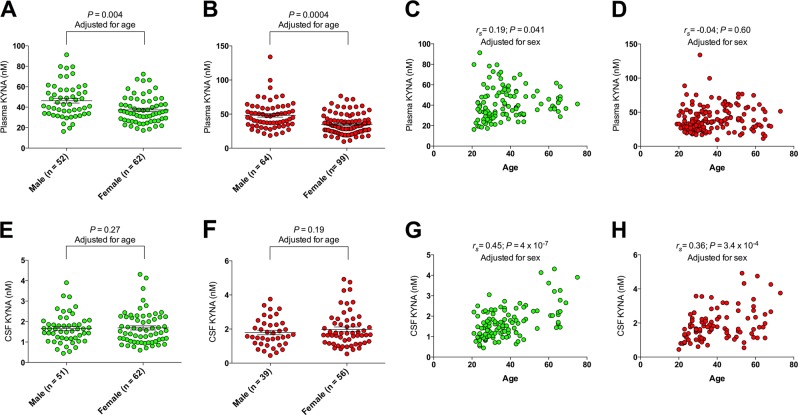
The effect of sex and age on peripheral and central kynurenic acid (KYNA) levels in healthy controls (HCs) and bipolar disorder (BD) subjects. **a** Plasma KYNA levels in female and male HCs. **b** Plasma KYNA levels in female and male bipolar subjects. **c** The effect of age on plasma KYNA levels in HCs. **d** The effect of age on plasma KYNA levels in bipolar subjects. **e** CSF KYNA levels in female and male HCs. **f** CSF KYNA levels in female and male bipolar subjects. **g** The effect of age on CSF KYNA levels in HCs. **h** The effect of age on CSF KYNA levels in bipolar subjects. Group comparisons in **a**, **b**, **e**, and **f** were performed using logistic regression models (age as covariate) with sex as dependent variable (0 = male, 1 = female). Correlation coefficients in **c**, **d**, **g**, and **h** are Spearman’s (*r*_s_) and derived from partial correlation analyses with sex as a covariate. All reported *P*-values are two-sided

Peripheral KYNA levels in patients with BD and HCs were measured in plasma from a total of 277 subjects (114 HCs and 163 subjects with either BD type I [*n* = 93] or II [*n* = 70]). Among the 116 males (42%) and 161 females the median age was 34 years (IQR = 17.5). More detailed demographics and clinical characteristics are given in Table S1. Female HCs, as well as female BD subjects, displayed lower plasma concentrations of KYNA than male HCs and male BD subjects (*β* = −0.04; *P* = 0.004, and *β* = −0.05; *P* = 5 × 10^−4^, respectively; logistic regression with male/female as dependent variable and age as covariate; Fig. 1a, b). Among HCs we observed a positive correlation between age and plasma levels of KYNA, while plasma KYNA levels were unaffected by age in BD (*r*_s_ = 0.19; *P* = 0.041, and *r*_s_ = 0.04; *P* = 0.60, respectively; partial Spearman correlation adjusted for sex; Fig. 1c, d). Smokers among HCs, as well as BD subjects, displayed plasma KYNA levels similar to non-smokers (*β* = −0.04; *P* = 0.51, and *β* = −0.01; *P* = 0.18, respectively; smoker/non-smoker as dependent variable and age/sex as covariates). Similar, body mass index (BMI) did not influence plasma KYNA levels in either HCs or BD subjects (*r*_s_ = 0.1; *P* = 0.15, *r*_s_ = 0.01; *P* = 0.92, respectively; adjusted for sex and age).

CSF concentrations of KYNA were measured in 113 HCs (51 males; 45%, and 62 females) and in 82 of the subjects with BD type I/II, together with another 11 BD I/II subjects who did not contribute with plasma samples (in total 94 BD subjects; 39 males and 55 females); see also Table S1). The median age in this sample was 36 years (IQR = 20.5). In contrast to plasma levels, male and female HCs, as well as BD subjects, displayed similar CSF KYNA levels (*β* = 0.33; *P* = 0.27, and *β* = 0.37; *P* = 0.19, respectively; male/female as dependent variable and age as covariates; Fig. 1e, f), while CSF concentration of KYNA increased by age in HCs as well as BD subjects (*r*_s_ = 0.45; *P* = 4.0 × 10^−7^, and *r*_s_ = 0.36; *P* = 3.4 × 10^−4^, respectively; adjusted for sex; Fig. 1g, h). We found no indications that smoking, or BMI, affected CSF KYNA levels in HCs or BD subjects (smoking: *β* = −0.02; *P* = 0.27, and *β* = 0.07; *P* = 0.80, respectively; smoker/non-smoker as dependent variable and age/sex as covariates, BMI: *β* = 0.14; *P* = 0.15, and *β* = 0.01; *P* = 0.92, respectively; adjusted for sex and age).

**Fig. 2:**
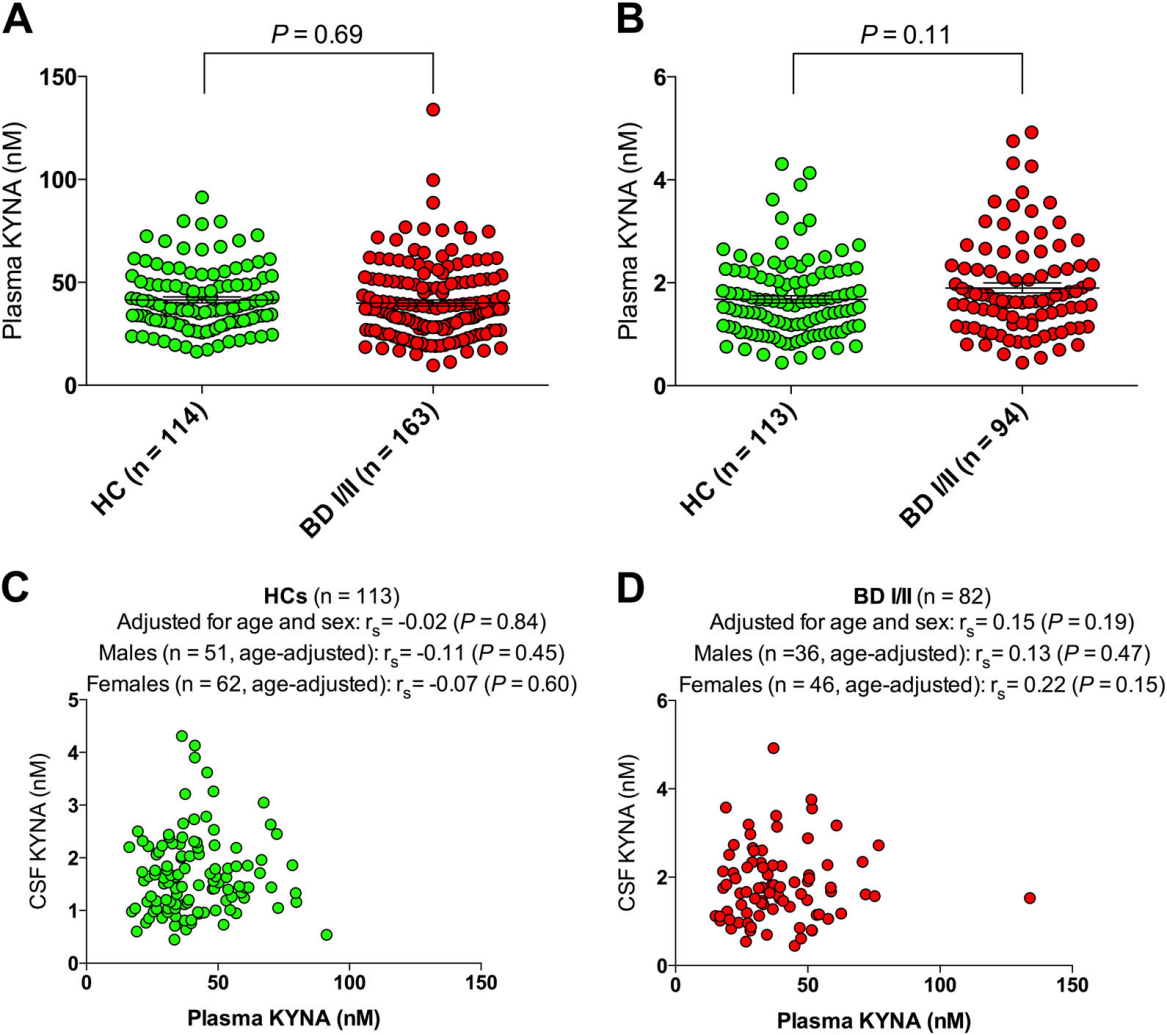
Peripheral and central kynurenic acid (KYNA) levels in healthy controls (HCs) and bipolar disorder (BD) subjects. **a** Plasma KYNA levels in HCs (median = 37.7 nM, IQR = 18.6) and BD I/II subjects (median = 37.0 nM, IQR = 23.2). **b** Cerebrospinal fluid (CSF) KYNA levels in HCs (median = 1.56 nM, IQR = 1.03) and BD I/II subjects (median = 1.66 nM, IQR = 1.16). **c** Correlations between plasma and CSF KYNA levels in HCs. **d** Correlations between plasma and CSF KYNA levels in BD I/II subjects. Group comparisons were performed using logistic regression models (age- and sex as covariates) with group as dependent variable (0 = HC, 1 = BD). Reported correlation coefficients (panel **c** and **d**) are Spearman’s (*r*_s_) and derived from partial correlation analyses with age and sex as covariates or partial correlation analyses with age as a covariate (stratified on sex). See also Fig. S1 and S2. All reported *P*-values are two-sided

BD subjects and HCs displayed similar plasma KYNA concentrations (HCs: median = 37.7 nM, IQR = 18.6, BD: median = 37.0 nM, IQR = 23.2), as well as CSF KYNA levels (HCs: median = 1.56 nM, IQR = 1.03, BD: median = 1.66 nM, IQR = 1.16), and age- and sex adjusted analyses revealed no significant differences in KYNA concentrations between HCs and BD subjects (Fig. 2a, b). Follow-up sensitivity analyses, excluding subjects with co-morbid somatic illness (adjusting for age and sex), or sex-stratified analyses (adjusting for age), revealed no significant differences in mean CSF KYNA levels between BD subjects and HCs (Fig. S1-S4).

Plasma and CSF KYNA measurements displayed no correlation in the whole sample (*r*_s_ = 0.04; *P* = 0.58; adjusted for age, sex, and case status), or in subgroup analyses stratifying on case status, or case status and sex (Fig. 2c, d, and Fig. S5). Excluding bipolar subjects with comorbid somatic illness again had no major impact on the relationship between CSF and plasma KYNA levels in BD subjects (data not shown), and analyses using age strata did not reveal any age dependent effects on a possible correlation between plasma and CSF measurements (20–39 years: *r*_s_ = 0.07; *P* = 0.42, 40–59 years: *r*_s_ = 0.21; *P* = 0.16, and 60–79 years: *r*_s_ = 0.12; *P* = 0.60; adjusted for sex and case status).

In bipolar subjects, we also studied dose-dependent effects of ongoing medications on plasma as well as CSF KYNA levels but without evidence of any significant effects on either plasma or CSF measurements of KYNA (Table S2).

### Peripheral and central KYNA levels in bipolar disorder—lifetime psychotic Symptoms and cognitive functioning

**Fig. 3:**
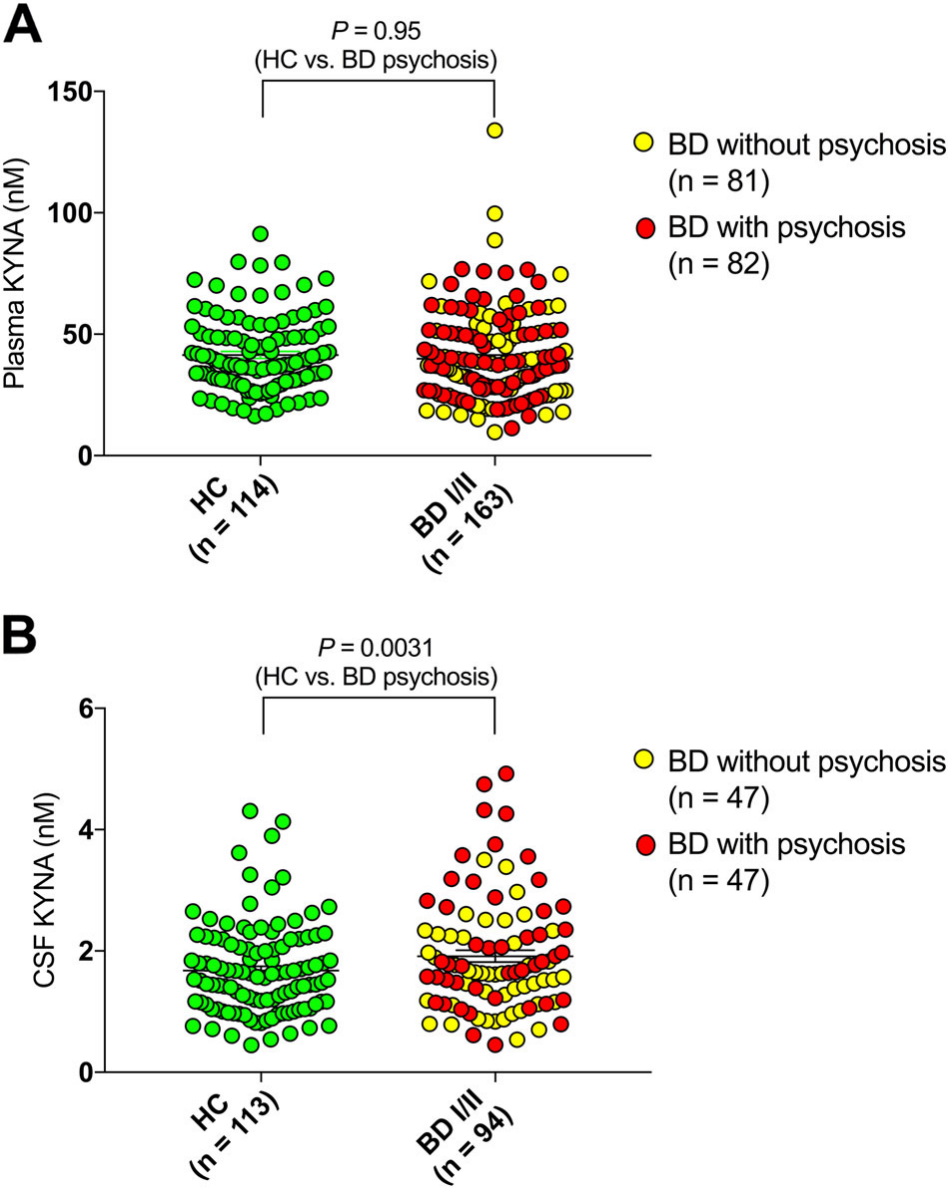
Peripheral and central kynurenic acid (KYNA) levels in bipolar disorder (BD) and healthy controls (HCs)—lifetime psychotic episodes. **a** Plasma KYNA levels HCs (median = 37.7 nM, IQR = 18.6) and bipolar disorder (BD) I/II subjects (median = 37.0 nM, IQR = 23.2). BD subjects with a history of psychotic episodes (median = 38.6 nM, IQR = 23.3), as well as BD subjects without such a history (median = 34.4 nM, IQR = 37.8), displayed plasma KYNA levels similar to HCs (adjusted for age and sex). **b** Cerebrospinal fluid (CSF) KYNA levels in HC (median = 1.56 nM, IQR = 1.03) and BD I/II subjects (median = 1.66 nM, IQR = 1.16). In sex and age adjusted analyses, BD subjects with a history of psychosis (median = 1.82 nM, IQR = 1.44) displayed increased CSF KYNA levels compared to HCs (*P* *=* 0.0031) as well as compared to BD subjects without a history of psychotic episodes (median = 1.57 nM, IQR = 1.02; BD psychosis vs. BD no psychosis; *P* = 0.0069). HCs and BD subjects without a history of psychotic episodes displayed similar CSF KYNA concentrations (*P* = 0.55). All reported *P*-values are two-sided and derived from logistic regression models with sex and age as covariates

In the bipolar cohort with available plasma KYNA concentrations 82 subjects had experienced psychotic episodes (defined as occurrence of hallucinations and/or delusions during a mood episode), i.e., 50%. As we previously have observed increased CSF KYNA concentrations in euthymic BD subjects with a history of psychosis^5,6^, we now compared plasma KYNA levels between this BD subgroup and HCs. Unlike previous results for CSF KYNA levels we observed no difference in mean KYNA plasma levels between this BD subgroup and HCs (adjusted for sex and age; Fig. 3a). Excluding subjects with comorbid somatic illness, or stratifying on sex, revealed no significant differences between patients and controls (Fig. S6 and S7).

Among subjects with CSF data, now using a substantially larger sample as in previous studies^5,6^, we observed significantly increased CSF KYNA levels in the 47 BD subjects (50%) with a history of psychosis (Fig. 3b). Similar results were obtained excluding subjects with comorbid somatic illness (Fig. S8) and sex-stratified analyses suggested effects of similar magnitude in females and males (Fig. S9).

Given our previous data showing increased CSF KYNA levels in euthymic bipolar subjects with set-shifting impairments^6^, we now also in a subset of the sample (*n* = 97) with available cognitive evaluations studied plasma KYNA levels in relation to set-shifting performance again using the Trail Making Test (TMT; Switching vs. Combined Number Letter Sequencing). In contrast to previous analyses of CSF KYNA concentrations, mean plasma KYNA concentrations did not differ between bipolar subjects who scored below the mean standard score of 10 and bipolar subjects scoring ≥ 10, and plasma KYNA displayed no correlation with TMT scores when treated as a continuous variable (Fig. S10).

### Peripheral and central KYNA levels in bipolar disorder—type I and II disorder

Among the cohort with available plasma, 93 subjects (57%) had a BD I diagnosis and 70 subjects a type II diagnosis. 75 of the bipolar I subjects (81%) had a history of psychosis while 7 (10%) of the BD II subjects had a history of psychosis (depressive episode with psychotic features). As with contrasting BD subjects based on psychosis (Fig. 3), we observed no difference in KYNA plasma levels between BD I and BD II subjects after adjustment for sex and age (Fig. S11).

In the cohort with CSF samples, 55 subjects (59%) were diagnosed with bipolar I (42 (76%) of these subjects had a history of psychosis), and 39 subjects had a bipolar II diagnosis (13% of these subjects had a history of psychosis). Unlike analyses dividing the sample into psychotic and non-psychotic subjects, we observed no significant association between CSF KYNA levels and subtype of bipolar disorder (Fig S11).

### Peripheral and central KYNA levels in bipolar disorder—current depressive symptoms

**Fig. 4:**
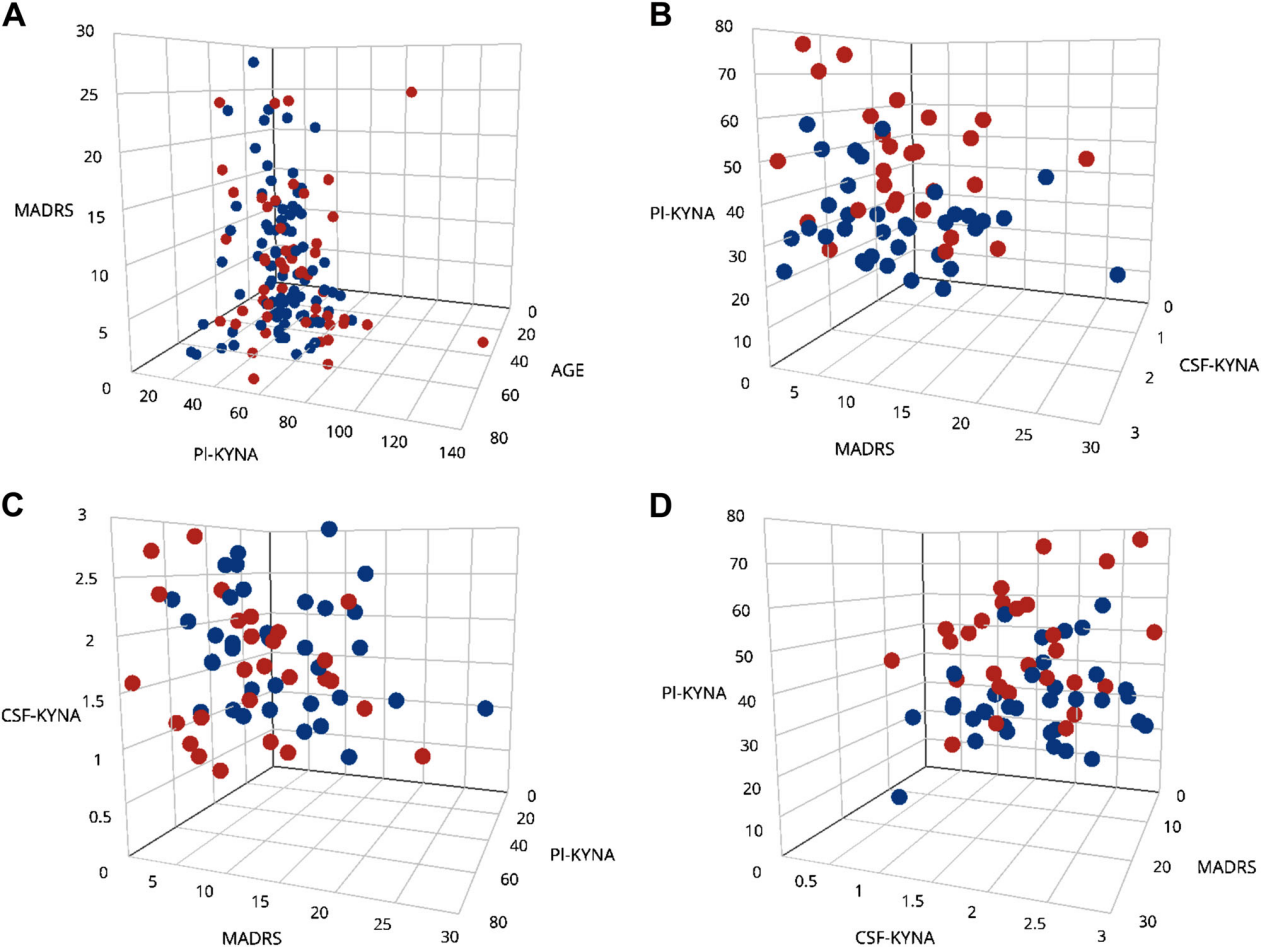
Peripheral and central kynurenic acid (KYNA) levels in bipolar disorder (BD)—ongoing depressive symptoms. **a** Plasma KYNA levels in healthy controls (HCs; median = 37.7 nM, IQR = 18.6) and BD subjects (median = 37.0 nM, IQR = 23.2) stratified on total MADRS score. In age and sex adjusted analyses, BD subjects with MADRS score > 4 (median = 33.0 nM, IQR = 14.2) displayed significantly decreased plasma KYNA levels compared to HCs (*P* = 0.046) although the decrease did not reach significance when comparing with BD subjects and MADRS score < 5 (median = 38.0 nM, IQR = 23.6; *P* = 0.070). **b** Cerebrospinal fluid (CSF) KYNA levels in HCs and BD subjects stratified on total MADRS score. Median (IQR) CSF KYNA levels in HCs: 1.56 nM (1.03), BD subjects with MADRS score < 5: 1.91 nM (1.54), and BD subjects with MADRS score > 4: 1.58 (0.93). In age and sex adjusted analyses, no significant difference in CSF KYNA levels could be observed between BD subjects with MADRS score > 4 and HCs (*P* = 0.34), while this group displayed decreased CSF KYNA levels compared to remaining BD subjects without ongoing depressive symptoms (*P* = 0.041). However, as BD subjects displaying depressive symptoms more seldom had a history of psychosis (36 vs. 57%) the difference in CSF KYNA concentration between the two BD groups defined by MADRS score was largely explained by distribution of psychosis (*β* = −0.62; *P* = 0.041 unadjusted vs. *β* = −0.54; *P* = 0.081 adjusted for psychosis). All *P*-values are two-sided and derived from logistic regression models with age and sex as covariates (and in the comparison of CSF KYNA levels between BD subjects also controlling for a history of psychosis, see above)

To assess current depressive symptoms in BD subjects, we used total score on MADRS at time of lumbar puncture and blood collection. Subjects with a total score < 5 (49%), were judged to be in complete remission regarding depression^29^. Excluding these subjects, the remaining BD subjects displayed significantly decreased plasma KYNA levels compared to HCs (adjusting for age and sex), although not reaching significance compared to BD subjects with MADRS score < 5 (Fig. 4a), or against HCs when excluding subjects with comorbid somatic illness (*β* = −0.02; *P* = 0.069, *n* = 171).

CSF KYNA levels were not significantly decreased in BD subjects with MADRS score > 4 (59%) comparing to HCs, although a significant decrease was observed comparing these subjects to the rest of the BD group (Fig. 4b). However, fewer subjects with remaining depressive symptoms had a history of psychosis (37 vs. 59%, respectively) explaining the observed decrease between the two BD groups divided on MADRS score (see figure legend for Fig. 4b). In agreement with our analyses using the complete BD group, we observed no correlation between plasma and CSF KYNA levels in the subgroup with remaining depressive symptoms (*r*_s_ = 0.09; *P* = 0.67; adjusted for age and sex).

### Peripheral and central KYNA levels in bipolar disorder—lifetime suicide attempt or self-harm

**Fig. 5:**
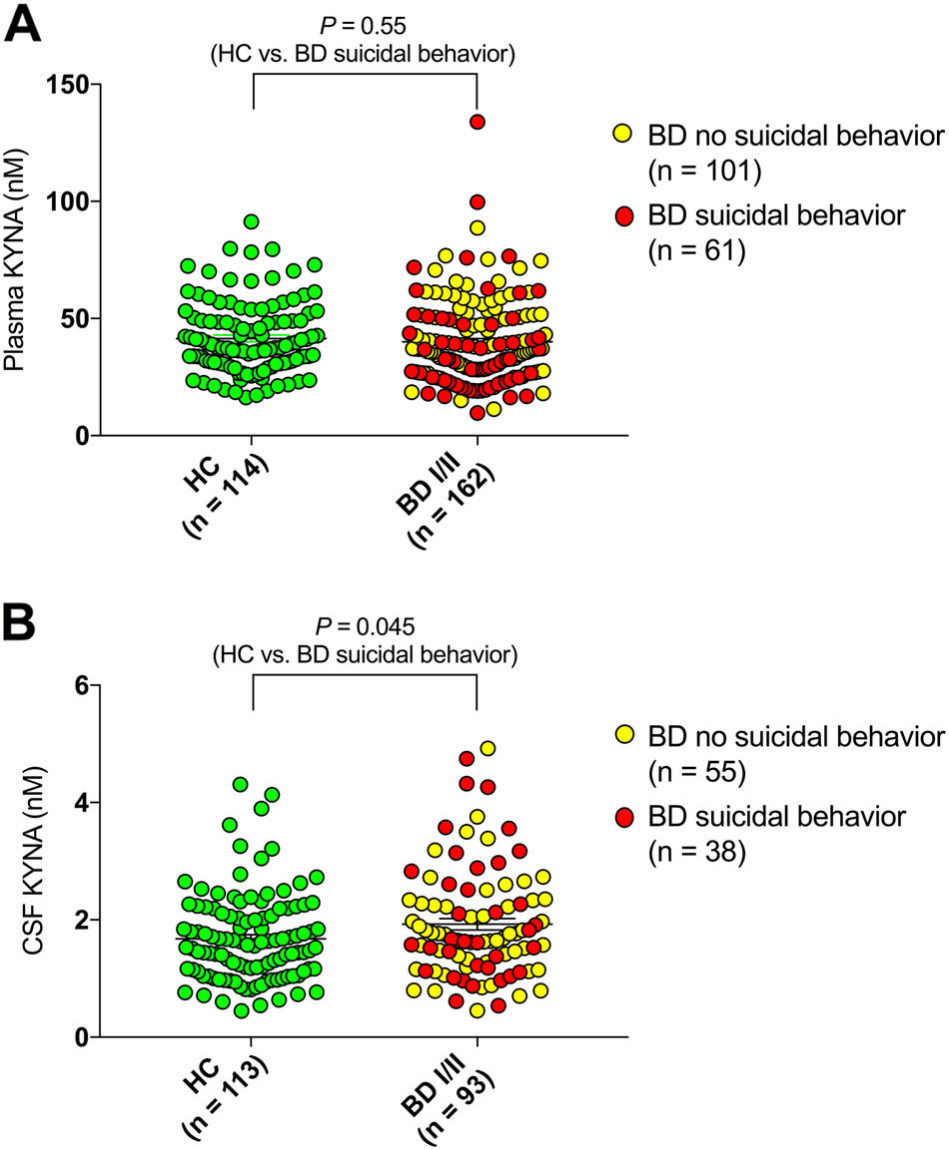
Peripheral and central kynurenic acid (KYNA) levels in bipolar disorder (BD) and healthy controls (HCs)—lifetime history of suicide attempt or self-harm. **a** Plasma KYNA levels in HCs (median = 37.7, IQR = 18.6) and BD subjects (median = 37.0, IQR = 23.2) stratified on a history of suicide attempt/self-harm (median for BD no suicide attempt/self-harm = 37,2; IQR = 21.2, median for BD suicide attempt/self-harm = 32.6; IQR = 24.5). Given the high number of females among BD subjects with a history of suicide attempt/self harm (70.5 vs. 55% in BD subjects with no such history) there was no significant differences in KYNA plasma concentrations for this group compared to HCs or BD without a history of suicide attempt/self-harm when adjusting for age and sex (BD suicide attempt/self-harm vs. BD no suicide attempt/self-harm: *P* = 0.57). **b** Cerebrospinal fluid (CSF) KYNA levels in HC (median = 1.56 nM, IQR = 1.03) and BD I/II subject (median = 1.66 nM, IQR = 1.16). BD subjects with a history of suicidal behavior (median = 1.63 nM, IQR = 1.67) displayed significantly increased CSF KYNA concentrations compared to HCs although not reaching significance compared to the smaller BD without a history of suicidal behavior group (median = 1.76 nM, IQR = 1.00; *P* = 0.33). All reported *P*-values are two-sided and derived from logistic regression models with sex and age as covariates

In the plasma cohort, 101 subjects had a lifetime history of suicide attempt or self-harm. This group did not display plasma KYNA levels that differed from HCs or bipolar subjects without such a history (Fig. 5a).

Thirty-eight subjects with CSF data had a lifetime history of suicide attempt or self-harm. These subjects displayed a slightly higher mean CSF KYNA compared to HCs (Fig. 5b) although the comparison to the smaller BD group without history of suicide attempt or self-harm did not reach significance (Fig. 5b). Distribution of lifetime psychotic symptoms was also similar between BD subjects with or without history of suicide attempt or self-harm (Fisher’s exact test; *P* = 1.0).

## Discussion

Our findings provide further evidence of a robust association between CSF levels of KYNA and psychotic BD, in agreement with our previous reports using smaller sample sizes^4,5^. In contrast, plasma KYNA concentrations were unchanged in BD subjects with a history of psychosis compared to HCs. These findings are in line with previous rodents studies supporting brain-specific regulation of KYNA dependent on factors such as glial energy metabolism and neuronal signals^27–30^, and suggest “on site” brain pathology as an important factor causing increased central KYNA levels in psychotic BD. Notably, symptomatology associated with increased CSF KYNA levels, i.e., delusions and hallucinations are also prominent features in schizophrenia and previous studies report similar increases in central KYNA levels in schizophrenia^2,6–8^. Important for future studies, our association analyses also revealed that age and sex influenced KYNA concentrations differently dependent on if measured in the periphery or in CNS. While females displayed lower plasma levels, sex did not influence central KYNA levels. Again, this implies brain-specific regulation of KYNA although the specific mechanisms remain elusive. Age displayed an even more complex influence on KYNA levels with healthy controls displaying an age-dependent increase in plasma and an even more pronounced effect in CSF, while bipolar subjects only displayed increasing CSF levels by age. It remains uncertain if the disease-specific lack of an age effect in plasma is due to confounding or is part of the pathophysiology as the mechanisms by which ageing effects the biosynthesis of the kynurenine metabolites is still largely unknown^30^.

We also observed decreased levels of plasma and CSF KYNA in BD with ongoing depressive symptoms in relation to HC, although not reaching significance when compared to non-depressed BD subjects. This supports the findings by Wurfel et al. and Birner et al.^16,18^. However, we observed no correlation between CSF and plasma KYNA concentrations in the whole sample or in the subset of BD subjects displaying ongoing depressive symptoms. By contrast, we also observed a significant increase in CSF KYNA levels in subjects with a lifetime history of suicidal behavior compared to HCs.

Several limitations in the current study should be highlighted. First, with our limited number of subjects displaying more severe depressive symptoms it cannot be excluded that BD subjects with ongoing moderate and severe depression display decreased plasma KYNA levels directly representing a pathophysiological mechanism driven by KYNA in the brain. Further, our collected clinical data did not separate suicide attempt and self-harm. Thus, it is possible that analyses restricted to suicide attempters would have given us other results. Finally, central and peripheral measurements of a larger set of metabolites in the kynurenine pathway are also warranted to provide a more comprehensive understanding of brain kynurenines under physiological as well as pathophysiological conditions. Regarding kynurenine, it is however worth mentioning that in human plasma kynurenine and KYNA concentration display a strong correlation^31^.

In summary, our data suggest that (1) KYNA in CSF, but not in plasma, represents a biomarker for lifetime psychotic episodes in BD, (2) peripheral KYNA levels do not predict central KYNA levels in healthy volunteers or in BD subjects. Thus, studies incorporating KYNA levels should include central rather than peripheral measurements to allow meaningful conclusions, and must correct for age-effects and sex-effects as well as current depressive symptoms. Finally, brain-specific pathological changes of the kynurenine metabolism in psychotic disorders may offer specific and novel drug targets.

## Supplementary information

Supplemental Material
