# Supplementary material for: Peripheral and central levels of kynurenic acid in bipolar disorder subjects and healthy controls
Source: Transl Psychiatry. 2019 Jan 29;9:37. doi: 10.1038/s41398-019-0378-9 (PMC6351610; doi:10.1038/s41398-019-0378-9)
Supplement: Supplementary file 1 — Supplemental Material [file 41398_2019_378_MOESM1_ESM.docx]

**Supplementary Information**

**Peripheral and central levels of kynurenic acid in bipolar disorder subjects and healthy controls**

Carl M. Sellgren^1,2,3,4^, Jessica Gracias^1,2,3,4^, Oscar Jungholm^1^, Roy H. Perlis^2,3^, Lilly Schwieler^1^, Mikael Landen^3^, Sophie Erhardt^1*^

^1^Department of Physiology and Pharmacology, Karolinska Institutet, Stockholm, Sweden, ^2^Center for Experimental Drugs and Diagnostics, Center for Genomic Medicine and Department of Psychiatry, Massachusetts General Hospital, Boston, MA, USA; ^3^Department of Psychiatry, Harvard Medical School, Boston, MA, USA; ^3^ Institute of Neuroscience and Physiology, Department of Psychiatry and Neurochemistry, The Sahlgrenska Academy, University of Gothenburg, Gothenburg and Mölndal, Sweden; ^4^ Stockholm County Council, Stockholm, Sweden.

*Corresponding author: [sophie.erhardt@ki.se](mailto:sophie.erhardt@ki.se), Dept. of Physiology and Pharmacology, Karolinska Institute, Nanne Svartz väg 2, 117 77 Stockholm, Sweden

Supplementary Tables

**Table S1**

|  | Healthy controls | Bipolar disorder (BD) | P-value^12^ |
| --- | --- | --- | --- |
|  | ***n* = 114** | ***n* = 163** |  |
| Age | 35 (28-44) | 34 (28-46) | 0.95 |
| Sex (male/female) | 52/62 | 64/99 | 0.32 |
| BMI^1^ | 23.3 (21.7-25.4) | 25.4 (22.3-27.8) | **0.001** |
| Smoking (yes/no) | 16/97^8^ | 50/99 | **0.0003** |
| Total MADRS^2^ score | 0 (0-2) | 5 (0-11)^9^ | **<0.0001** |
| GAF-S^3^ | 80 (75-85) | 68 (60-75) | **<0.0001** |
| GAF-F^4^ | 80 (75-85) | 68 (60-72) | **<0.0001** |
| Psychosis (yes/no) | n/a | 82/81 | n/a |
| BD type I/II | n/a | 93/70 | n/a |
| Suicidal behavior (yes/no)^5^ | n/a | 61/101^10^ | n/a |
| TMT^6^ scaled contrast score | n/a | 9 (8-11)^11^ | n/a |
| CGI-BP^7^ | n/a | 4 (4-5) | n/a |

^1^Body mass index

^2^Montgomery-Åsberg Depression Rating Scale

^3^Global Assessment of Functioning; Symptoms

^4^Global Assessment of Functioning; Functioning

^5^Lifetime history of suicide attempt or self-harm

^6^Trail Making Test (D-KEFS)

^7^Clinical Global Impression - Bipolar

^8^Data missing for 1 subject

^9^Data missing for 23 subjects

^10^Data missing for 1 subject

^11^Data missing for 66 subjects

^12^P-values derived from Mann-Whitney U-test except sex and smoking (Fisher’s exact test)

**Table S2**

| Drug | Plasma KYNA | | | CSF KYNA | | |
| --- | --- | --- | --- | --- | --- | --- |
|  | *n* | *r_s_*^1^ (P) | Range in mg  (min – max) | *n* | *r_s_* (P) | Range in mg  (min – max) |
| Lithium | 98 | -0.014 (0.89) | 42-294 | 30 | 0.017 (0.93) | 42-294 |
| Divalproex | 16 | -0.43 (0.72) | 300-1600 | 11 | -0.092 (0.78) | 300-1600 |
| Olanzapine | 12 | 0.31(0.34) | 2.5-20 | 7 | -0.41 (0.43) | 2.5-20 |
| Quetiapine | 15 | 0.23 (0.41) | 25-1000 | 9 | 0.15 (0.70) | 25-1000 |
| Lamotrigine | 20 | -0.35 (0.13) | 25-400 | 12 | 0.042 (0.90) | 25-400 |

^1^Spearman correlation coefficient

Supplementary Figures

**
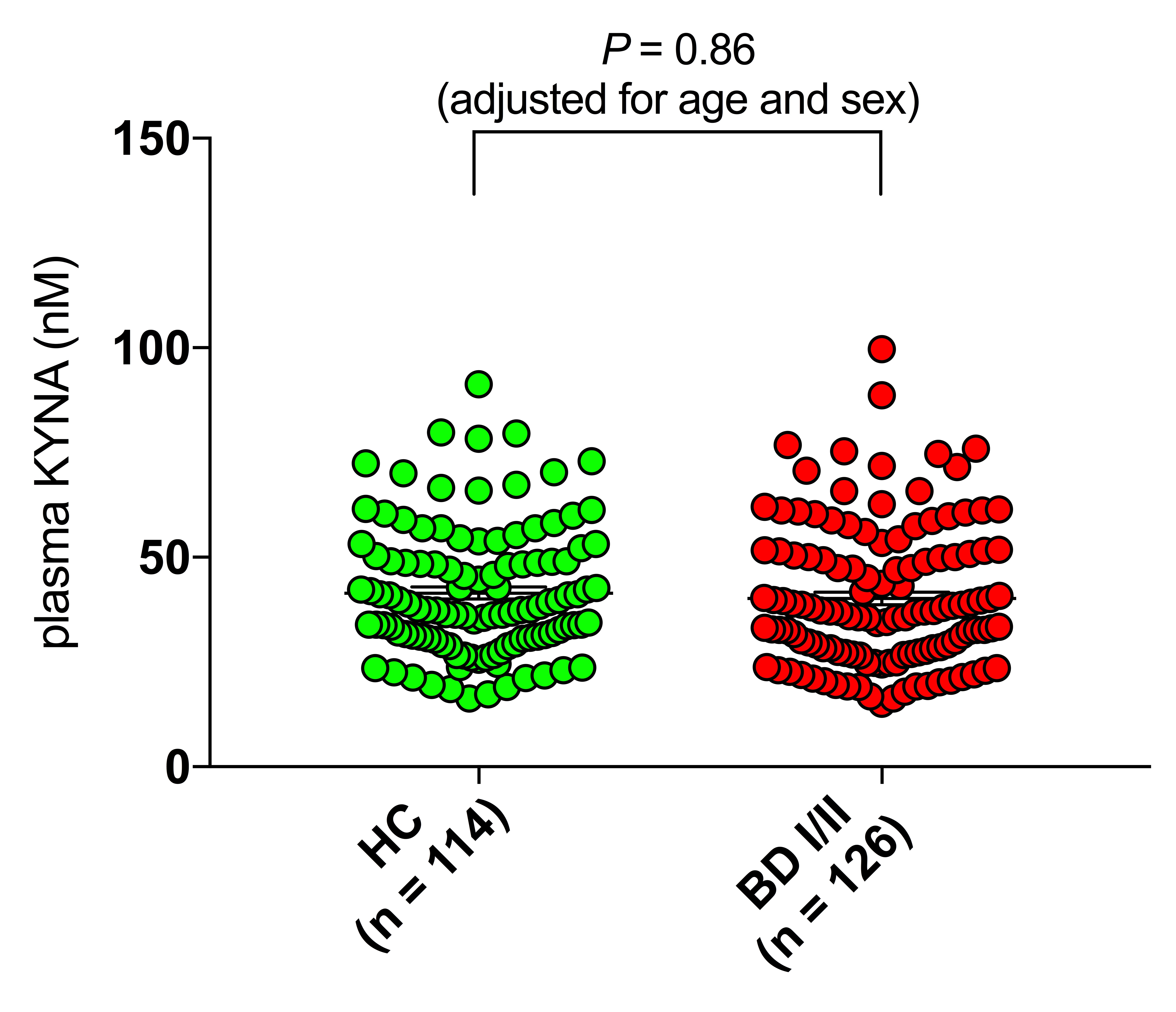
**

Figure S1

Plasma kynurenic acid (KYNA) levels in healthy controls (HCs) and bipolar disorder (BD) I/II subjects. For comparison to **Fig. 2A** this analysis excludes subjects with comorbid somatic illness (see ‘Study population’ in ‘Methods and materials’). Group comparison was performed using logistic regression models (age- and sex as covariates) with group as dependent variable (0 = HC, 1 = BD). Error bars represent unadjusted mean ± SEM. Reported p-value is two-sided.


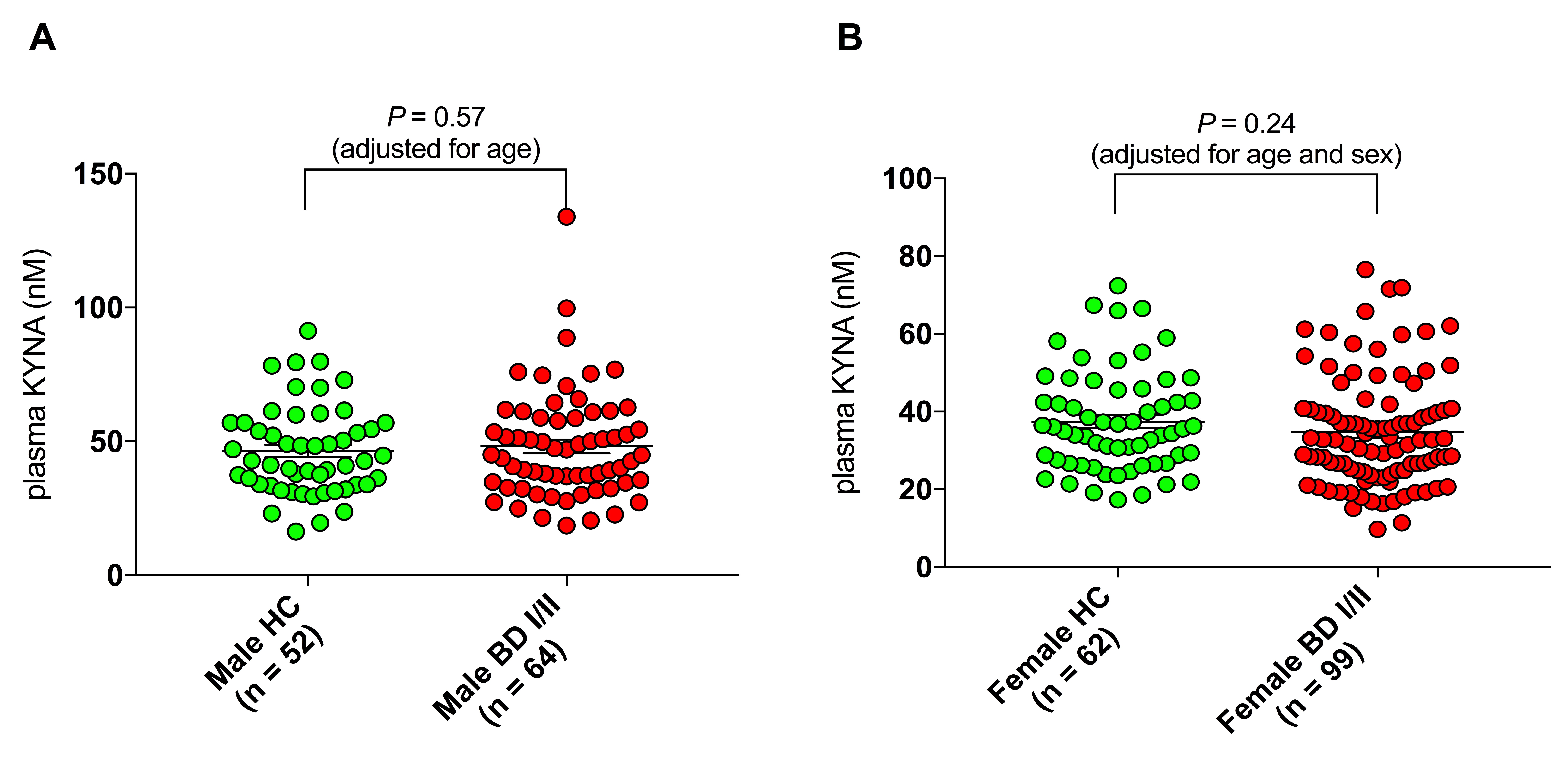


Figure S2

Plasma kynurenic acid (KYNA) levels in healthy controls (HCs) and bipolar disorder (BD) patients stratified on sex. (**A**) Plasma KYNA levels in Male HCs and BD type I/II subjects. (**B**) Plasma KYNA levels in female HCs and BD type I/II subjects. Group comparisons were performed using logistic regression models (age as a covariate) with group as dependent variable (0 = HC, 1 = BD). Error bars represent unadjusted mean ± SEM. Reported p-values are two-sided.

**
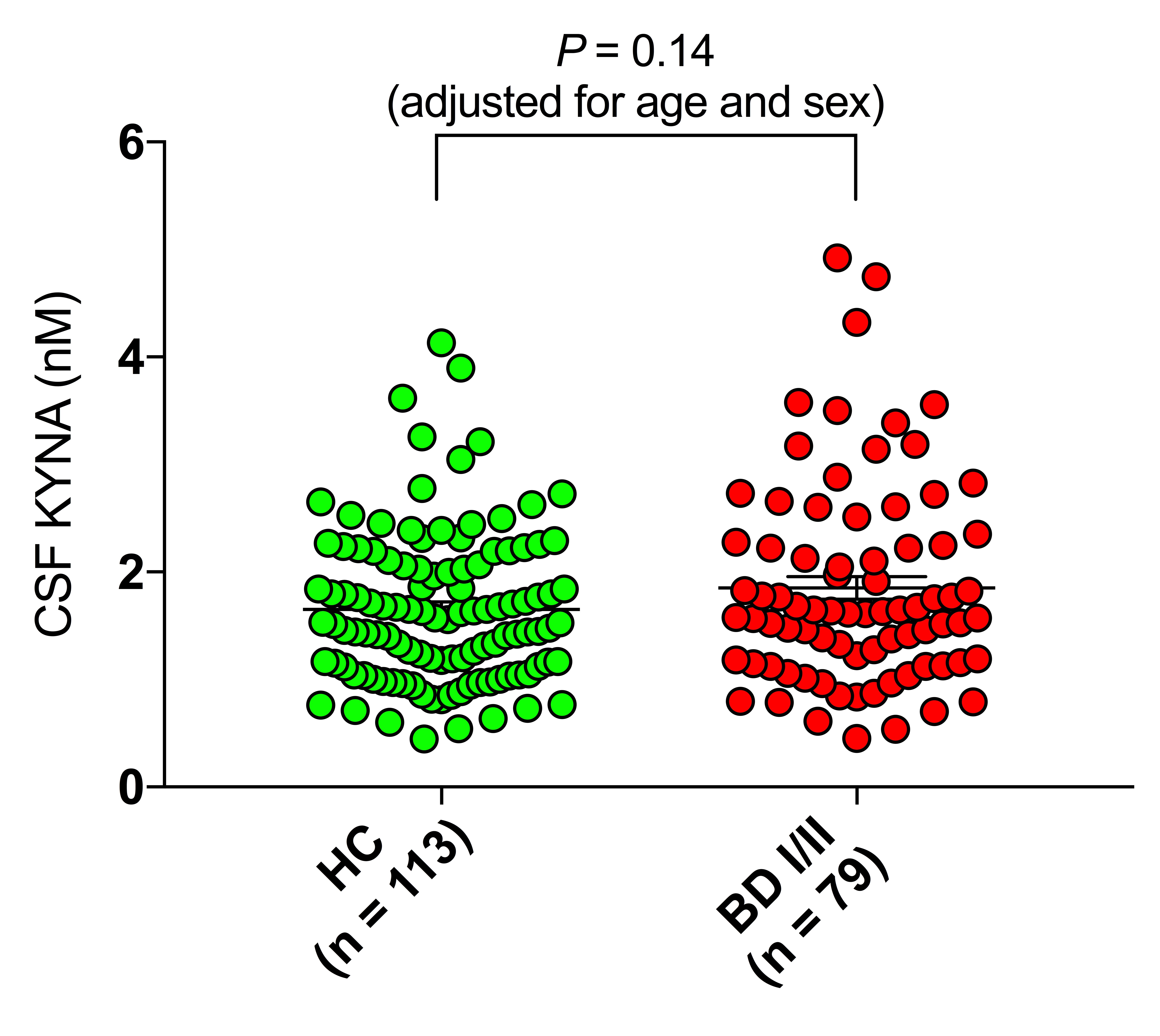
**

Figure S3

Cerebrospinal fluid (CSF) kynurenic acid (KYNA) levels in healthy controls (HCs) and bipolar disorder (BD) type I/II subjects. For comparison to **Fig. 2B** this analysis excludes subjects with comorbid somatic illness (see ‘Study population’ in ‘Methods and materials’). Group comparison was performed using logistic regression models (age- and sex as covariates) with group as dependent variable (0 = HC, 1 = BD). Error bars represent unadjusted mean ± SEM. Reported p-value is two-sided.

**
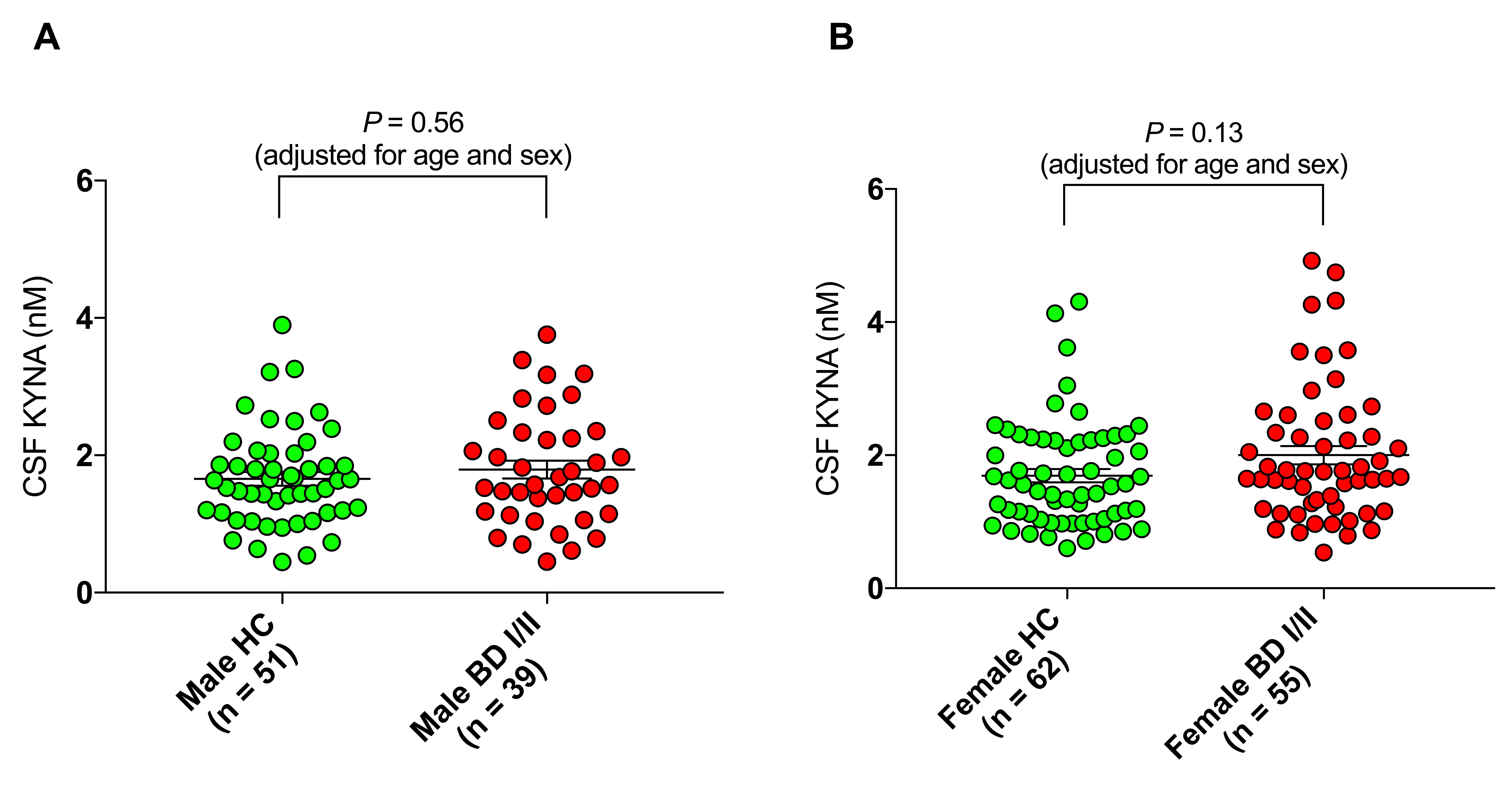
**

Figure S4

Cerebrospinal fluid (CSF) kynurenic acid (KYNA) levels in healthy controls (HCs) and bipolar disorder (BD) patients stratified on sex. (**A**) CSF KYNA levels in Male HCs and BD type I/II subjects. (**B**) CSF KYNA levels in female HCs and BD type I/II subjects. Group comparisons were performed using logistic regression models (age as a covariate) with group as dependent variable (0 = HC, 1 = BD). Error bars represent unadjusted mean ± SEM. Reported p-values are two-sided.

**
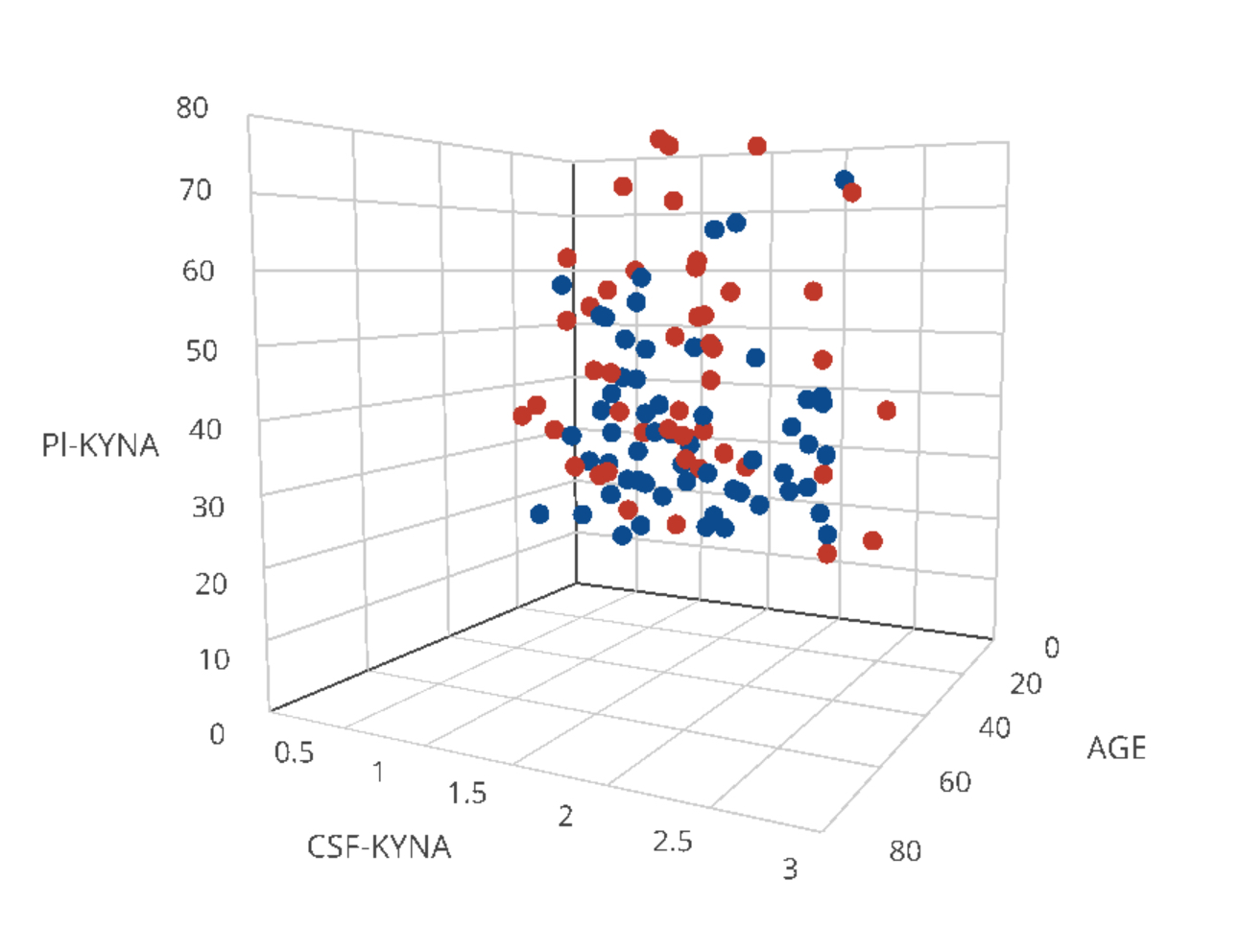

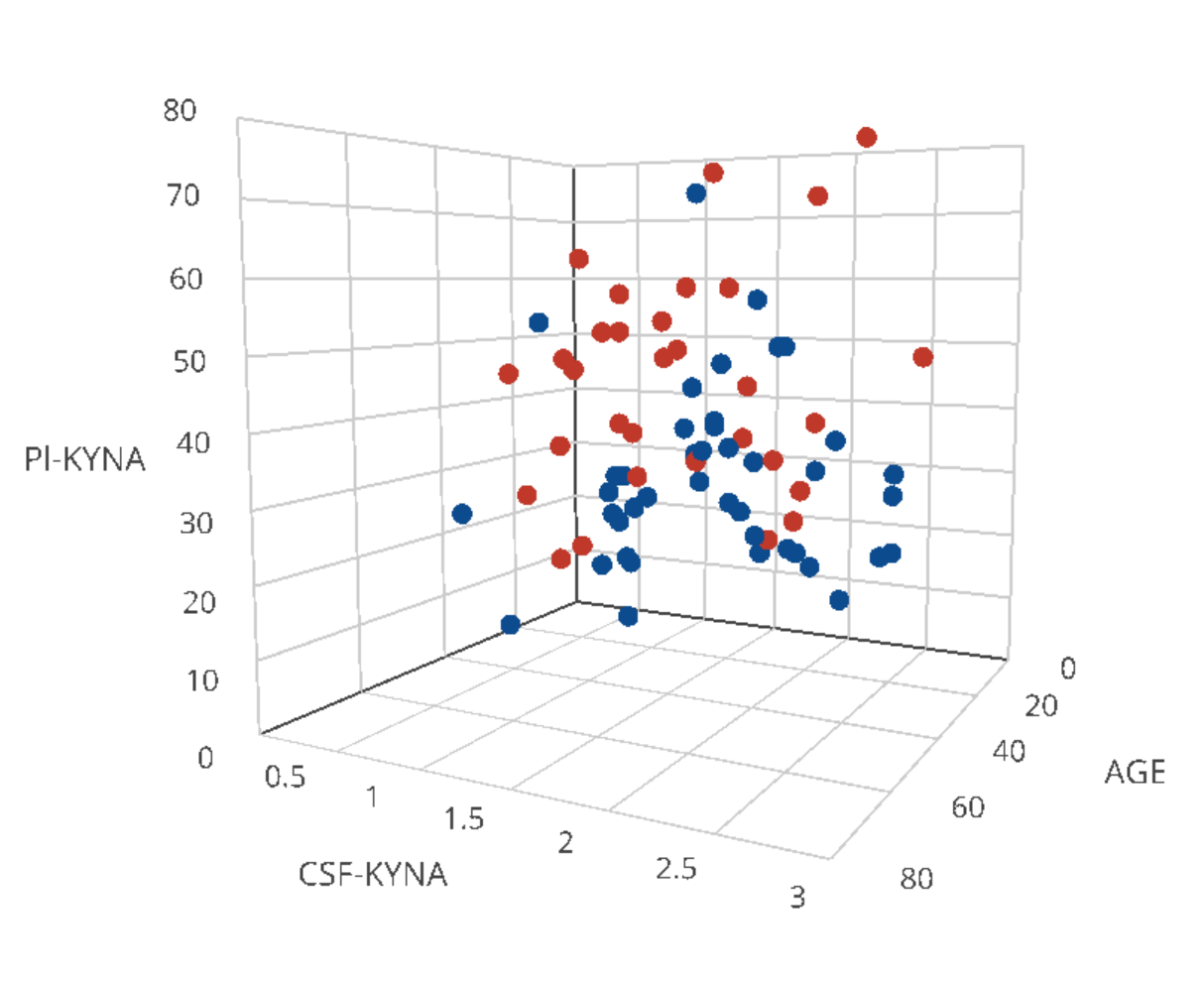
**

Figure S5

3D scatter plots of plasma (Pl), cerebrospinal fluid (CSF) KYNA levels, and age in healthy controls (**A**) and in bipolar disorder I/II patients (**B**). Red dots = males, and blue dots = females. Age and sex (red vs. blue), adjusted for in partial correlation (Spearman) analyses (see **Fig. 2C** and **2D)**.


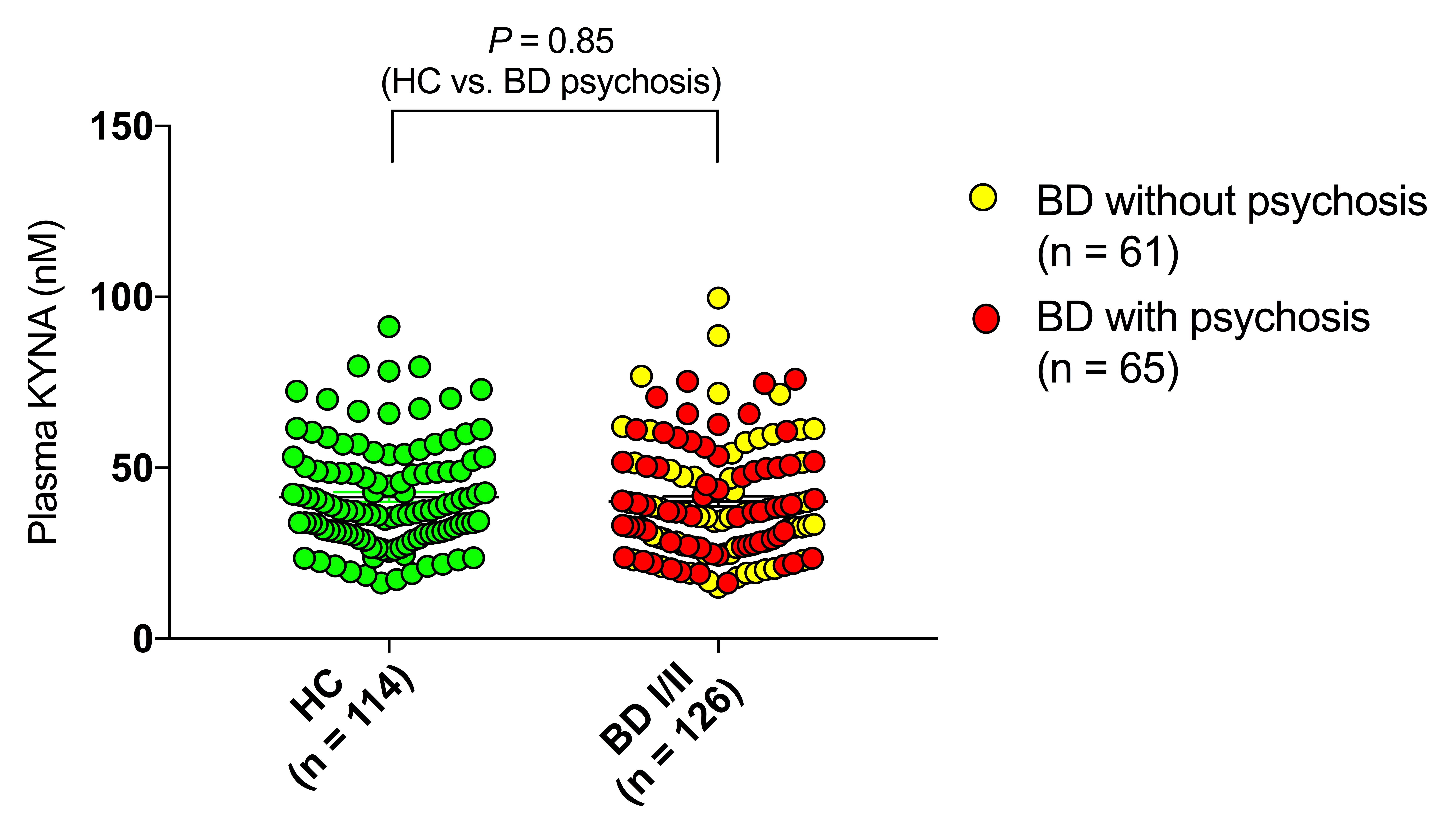


Figure S6

Plasma kynurenic acid (KYNA) levels in healthy controls (HCs) and bipolar disorder (BD) I/II subjects. BD subjects with a history of psychotic episodes (marked in red), as well as BD subjects without such a history (marked in yellow). For comparison to **Fig. 3A** this analysis excluded subjects with comorbid somatic illness (see ‘Study population’ in ‘Methods and materials’). All reported p-values are two-sided, derived from logistic regression models with sex and age as covariates, and represent comparisons between HCs and BD subjects with psychosis. No significant mean differences were observed between HCs and BD subjects without such a history as well as between the psychotic and non-psychotic BD group. Error bars represent mean ± SEM.


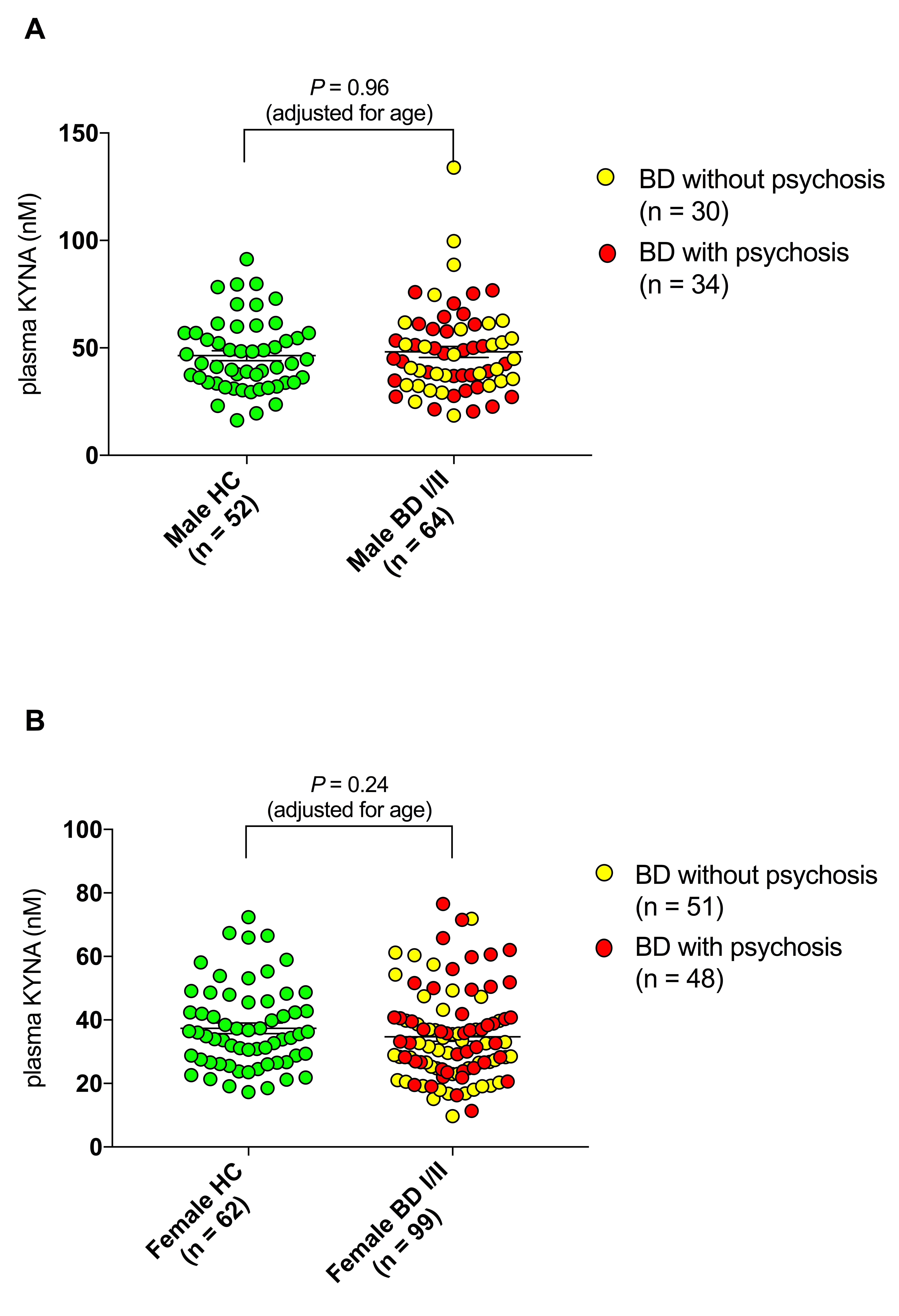


Figure S7

Plasma kynurenic acid (KYNA) levels in bipolar disorder (BD) subjects type I/II with or without a history of psychotic episodes and stratified on sex. (**A**) Plasma KYNA levels in males, and (**B**) plasma KYNA levels in female HCs and BD type I/II subjects. All reported p-values are two-sided, derived from logistic regression models age as covariates, and represent comparisons between HCs and BD subjects with psychosis. No significant mean differences were observed between HCs and BD subjects without such a history as well as between the psychotic and non-psychotic BD group. Error bars represent mean ± SEM.


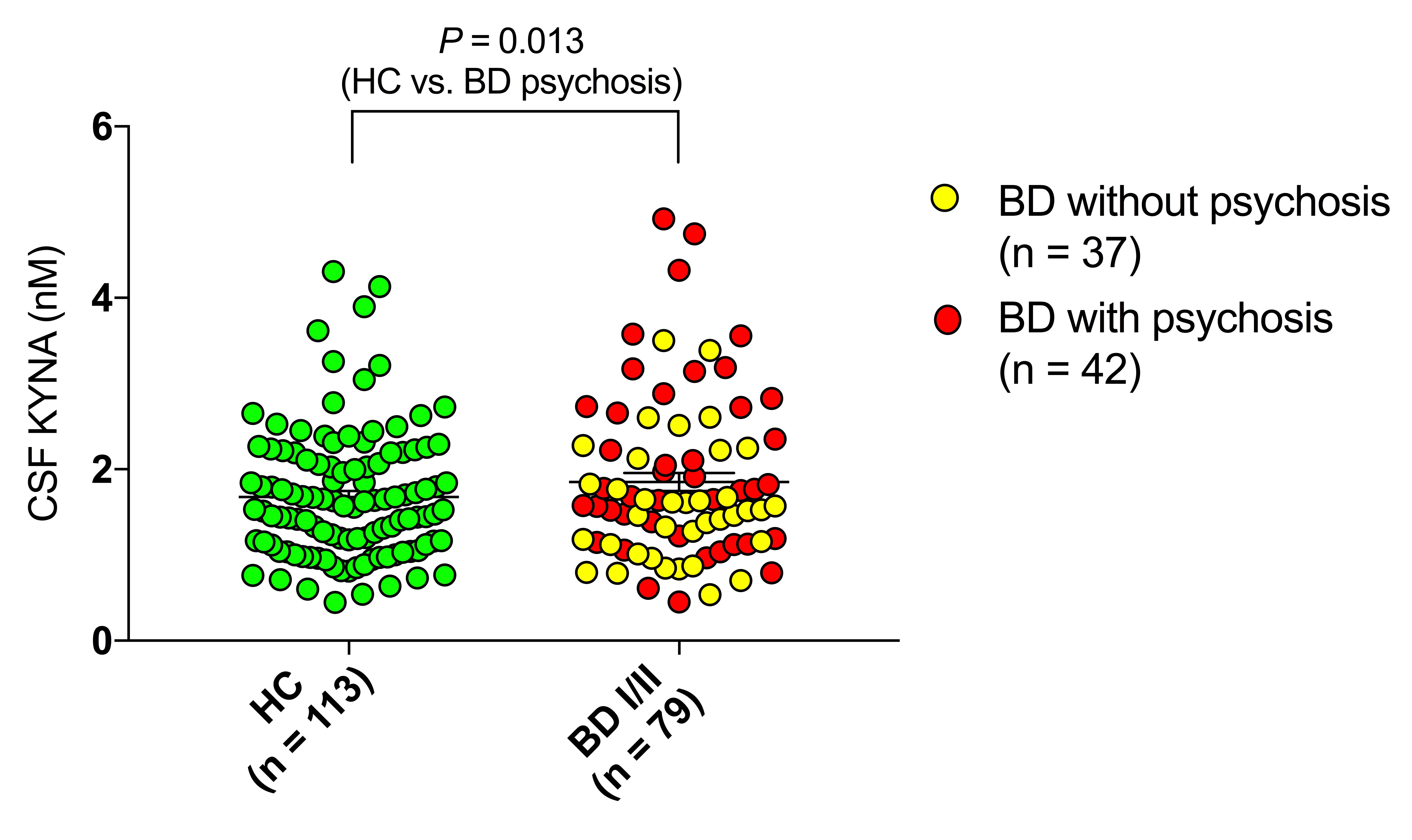


Figure S8

Cerebrospinal fluid (CSF) kynurenic acid (KYNA) levels in healthy controls (HCs) and bipolar disorder (BD) I/II subjects. BD subjects with a history of psychotic episodes (marked in red), as well as BD subjects without such a history (marked in yellow). For comparison to **Fig. 3B** this analysis excluded subjects with comorbid somatic illness (see ‘Study population’ in ‘Methods and materials’). Reported p-value is two-sided, derived from logistic regression models with sex and age as covariates, and represent comparisons between HCs and BD subjects with psychosis. No significant mean differences were observed between HCs and BD subjects without such a history while psychotic BD displayed significantly increased CSF KYNA levels compared to non-psychotic BD subjects (*P* = 0.029). Error bars represent mean ± SEM.





Figure S9

Cerebrospinal fluid (CSF) kynurenic acid (KYNA) levels in bipolar disorder (BD) subjects type I/II with or without a history of psychotic episodes and stratified on sex. (**A**) CSF KYNA levels in males, and (**B**) CSF KYNA levels in female HCs and BD type I/II subjects. All reported p-values are two-sided, derived from logistic regression models age as covariates, and represent comparisons between HCs and BD subjects with psychosis. Error bars represent mean ± SEM.


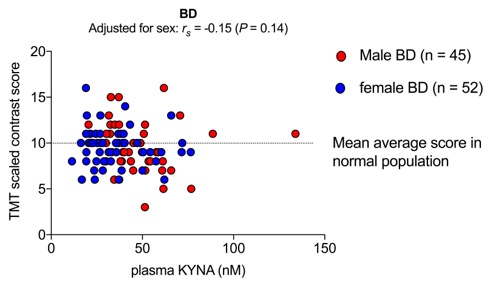


Figure S10

Peripheral kynurenic acid (KYNA) levels in bipolar disorder (BD) subjects – set-shifting performance. In the BD sample with scaled contrast score from trail making test (TMT, from D-KEFS, n = 97), we observed no correlation between TMT score and plasma KYNA levels. Data was analyzed using partial correlation analyses (Spearman’s correlation coefficient) with sex as covariates. Reported p-value is two-sided.

**
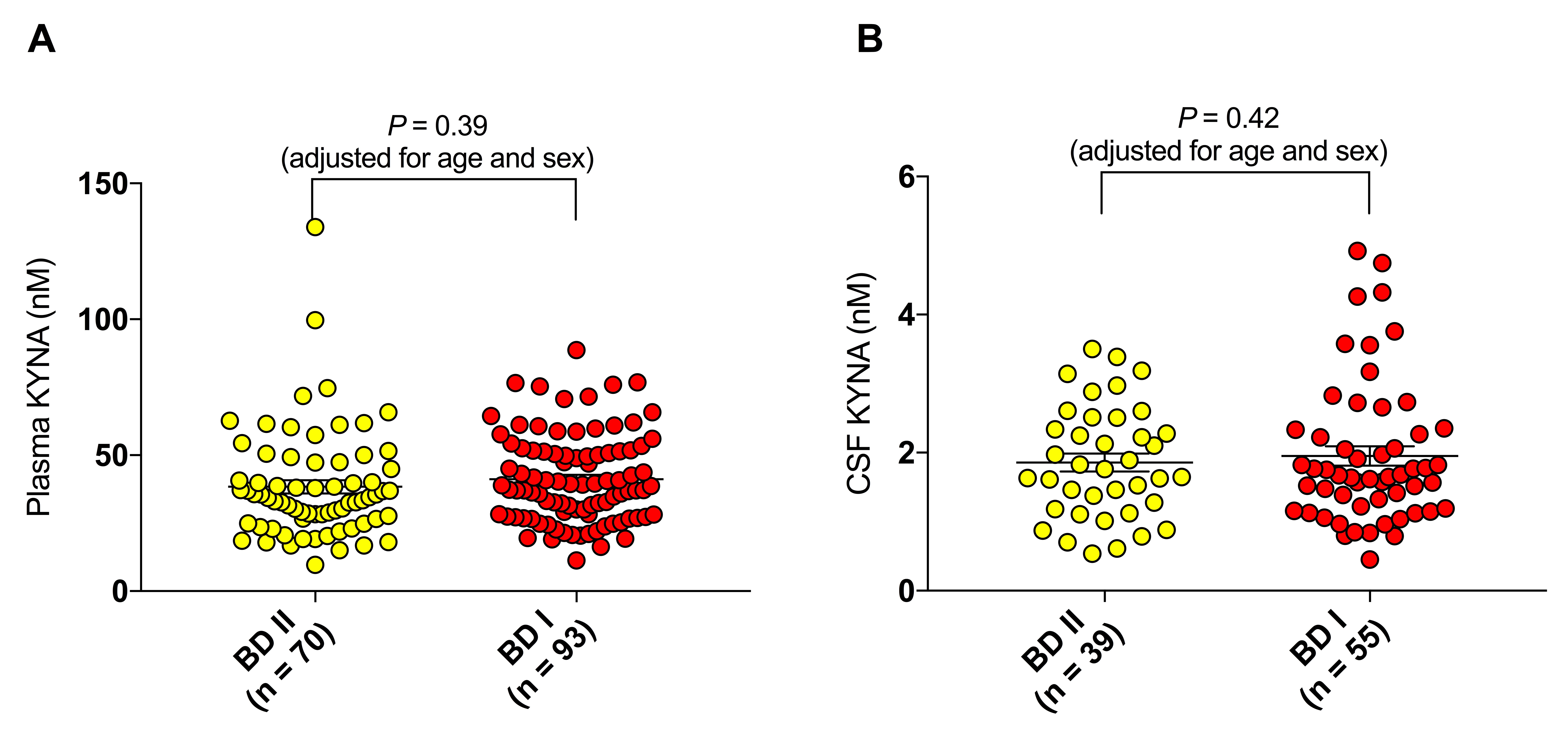
**

Figure S11

Plasma and cerebrospinal fluid (CSF) kynurenic acid (KYNA) levels in bipolar disorder (BD) subjects type I and II (**A**) Plasma KYNA levels in type I and II subjects, and (**B**) CSF KYNA levels in in type I and II subjects. All reported p-values are two-sided, derived from logistic regression models with age and sex as covariates. Error bars represent mean ± SEM.
